# Deep Eutectic Solvents for Ammonia Capture and Detection: Molecular Design, Absorption Mechanisms, and Analytical Applications: A Brief Review

**DOI:** 10.3390/ijms27188206

**Published:** 2026-09-15

**Authors:** Balzhan Satanova, Dinara Kalmanova, Aizhan Zhexembayeva, Omirzak Abdirashev, Aisulu Abuova, Fatima Abuova, Yerbolat Kalpakov, Almaz Orymbetov, Marina Konuhova, Elena Popova, Anatoli I. Popov

**Affiliations:** 1 Institute of Physical and Technical Sciences, L. N. Gumilyov Eurasian National University, Astana 010000, Kazakhstan; satanova_bm_2@enu.kz (B.S.); kalmanova_dm@enu.kz (D.K.); abuova_au@enu.kz (A.A.); abuova_fu@enu.kz (F.A.); popov@latnet.lv (A.I.P.); 2 School of Intelligent Systems, Astana IT University, EXPO C1, Mangilik El 55/11, Astana 010000, Kazakhstan; y.kalpakov@astanait.edu.kz; 3 National Laboratory Astana, Nazarbayev University, Astana 010000, Kazakhstan; almaz.orymbetov@nu.edu.kz; 4 Institute of Solid State Physics, University of Latvia, LV-1063 Riga, Latvia; marina.konuhova@cfi.lu.lv; 5 Ventspils International Radio Astronomy Centre, Ventspils University of Applied Sciences, LV-3601 Ventspils, Latvia; 6 Department of Physics, University of Maryland, College Park, MD 20742-4111, USA

**Keywords:** deep eutectic solvents (DESs), ammonia (NH_3_) capture, molecular design, absorption mechanisms, gas sensing and detection, green analytical chemistry, hydrogen bonding, solubility thermodynamics, gas separation (NH_3_/CO_2_ selectivity), natural deep eutectic solvents (NADES), microextraction (AALLME/DLLME), ab initio molecular dynamics (AIMD)

## Abstract

Ammonia (NH_3_) is a valuable resource for agriculture and a promising carbon-neutral hydrogen carrier, but current industrial emissions result in excessive environmental/health impacts. Popular approaches to removal—such as acid/water scrubbing and ionic liquids—are limited due to solvent volatility, costly regeneration, secondary salt wastes, or costly synthesis. Deep eutectic solvents (DESs) represent a sustainable and task-specific alternative, characterized by low vapor pressure and the capacity for straightforward, low-cost chemical customization. This review presents a digest of literature synthesis combining molecular design strategies, thermodynamic/atomistic mechanisms of absorption, process-scale modeling, and sensing applications for NH_3_, together with a classification of key DES synthesis protocols, including protic, multiacid/weak acid, azole-based, non-halide, supramolecular host–guest, and metal-coordinated systems. These synthesis strategies are designed to optimize absorption capacity, selectivity for NH_3_/CO_2_, transfer efficiency, and ease of regeneration. Molecular dynamics simulations and spectroscopies have been examined to elucidate the overall mechanism of absorption for a two-phase system (specific H-bonding, followed by weak physical dissolution), which dominates within DESs using specific atomistic interactions (hydroxyl-, amino-, or ammonium-residue) and van der Waals forces. Emerging sensing methods of DES have been briefly investigated (microextraction techniques: AALLME, DLLME; mobile phone colorimetry; and chemoresistive sensors). This work highlights the need for closer alignment between the atomistic models and the design of the sensor.

## 1. Introduction

Global demand for ammonia (NH_3_) is substantial, since NH_3_ is a principal component of nitrogen fertilizers. Reactive nitrogen—of which NH_3_ is a key product—has been as fundamental to the Agricultural Revolution as fossil-derived carbon was to the Industrial Revolution [1,2,3,4,5,6,7,8]. NH_3_ also contains a higher hydrogen mass fraction (17.6 wt%) than is typically achievable in competing hydrogen carriers, and its comparatively low liquefaction temperature gives it favorable storage and transport properties. This has attracted considerable research interest in NH_3_ as an energy and hydrogen carrier in its own right [9,10,11,12,13,14,15,16,17,18]. World production and consumption of NH_3_ are therefore expected to continue increasing [19].

This expansion carries substantial environmental costs. NH_3_ is corrosive to pipelines and industrial equipment [2,3] and is a known contributor to atmospheric fog and haze [2,3]. Large-scale NH_3_ emissions during chemical and fertilizer production contribute to exceedances of PM2.5 air quality standards, and the atmospheric oxidation of NH_3_ produces NO_x_, increasing atmospheric acidity [9,20,21,22,23,24,25,26,27]. Efficient removal and recycling of NH_3_ from industrial exhaust gas is therefore both an environmental necessity and an economic opportunity, since recovered NH_3_ retains value as a raw material [2].

Conventional NH_3_ removal from tail gas relies principally on water or inorganic acid scrubbing [9]. Both approaches have specific drawbacks: water exhibits high volatility and comparatively low NH_3_ absorption capacity, whereas the strong acid-base interaction between an acidic absorbent and NH_3_ is essentially irreversible and generates large volumes of low-value ammonium salt wastewater [2,3]. More broadly, commercial gas-scrubbing processes share common limitations—volatile solvents, generation of hazardous byproducts, and high regeneration energy demands [28,29,30,31,32,33,34,35,36,37,38]. Ionic liquids (ILs) have consequently been explored as an alternative class of NH_3_ absorbents, offering negligible vapor pressure, excellent thermal stability, and tunable cation–anion pairing [3,4], but their complex, costly synthesis and poor biodegradability have limited their adoption in practical NH_3_-capture applications [3,4].

Deep eutectic solvents (DESs) have emerged as a promising alternative, retaining many of the favorable properties of ILs—low volatility, wide liquid range, and tunable composition—while being prepared far more simply and economically by direct mixing of a hydrogen-bond acceptor (HBA) and a hydrogen-bond donor (HBD) without complex ionic synthesis [4,10]. DESs are further regarded as designable, task-specific green solvents with more favorable biocompatibility than conventional organic or acidic absorbents [23]. These “green” and “low-cost” characterizations should not, however, be applied uncritically to DESs as a class: component toxicity, biodegradability, and cost vary substantially with HBA/HBD chemistry, and some DESs incorporate metal salts, halide-containing components, or synthetic macrocyclic hosts that are neither inexpensive nor unambiguously benign (Section 9.4 discusses this trade-off further). On this basis, a substantial and growing body of literature has explored diverse DES chemistries for NH_3_ capture, including protic systems formed from amine hydrochloride salts and ammonium thiocyanate, azole- and phenol-based HBDs, supramolecular architectures incorporating macrocyclic hosts such as pillar[5]arene, and ternary systems incorporating metal centers—several of which report a higher NH_3_ absorption capacity than many functionalized ILs [4,9,18,19,20,21,22].

Despite this progress, the molecular-level mechanism of a NH_3_-DES interaction has been explored only sparsely, even though a detailed mechanistic understanding is necessary to move the DES design from an empirical to a rational basis [10]. The mechanistic studies that do exist indicate that NH_3_ absorption by DESs proceeds mainly via two pathways: an initial stage of hydrogen-bonding interaction at specifically designed donor sites (hydroxyl groups, amine groups, and ammonium cations), followed by van der Waals-dominated physical dissolution into the solvent matrix once the primary interaction sites are saturated [9,10]. Both ILs and DESs have been reviewed extensively as NH_3_ absorbents [19,25] and, more broadly, as absorbents for hazardous gases, including CO_2_, SO_x_, NO_x_, and H_2_S [38]. However, the relationship between DES molecular design, absorption mechanism, and NH_3_ sensing and analytical application has not previously been synthesized in a single review. This work addresses that gap by reviewing DES design strategies for NH_3_ capture, the underlying atomistic and thermodynamic mechanisms, and their emerging applications in NH_3_ sensing and analytical determination.

The evolution of DES research for NH_3_ capture over the past decade traces a clear trajectory from empirical formulation to rational design. Early studies relied on screening conventional HBA/HBD pairs, primarily choline chloride with urea or ethylene glycol, to identify systems with appreciable NH_3_ capacity, guided largely by intuition rather than predictive criteria. The subsequent expansion to protic salts, azole heterocycles, multiple weak-acid sites, and metal-containing formulations marked a transition toward targeted molecular engineering, wherein absorption performance is deliberately optimized through systematic tuning of HBD acidity, hydrogen-bond density, and supramolecular pre-organization. Concurrently, atomistic simulations have transformed mechanistic understanding from speculative inference to quantitative resolution of solvation–shell structures and interaction energies, while the recent emergence of DES-based sensing platforms signals a further conceptual shift: from DESs as passive absorbents to DESs as active recognition elements in analytical devices. This review synthesizes these three evolutionary threads, such as molecular design, mechanistic elucidation, and sensing integration, to map the current landscape and identify the critical gaps that define the next frontier.

## 2. Deep Eutectic Solvent Design Strategies for NH_3_ Capture

### 2.1. Protic DESs with Acidic/Hydrogen-Bond Donor Sites

**Figure 1:**
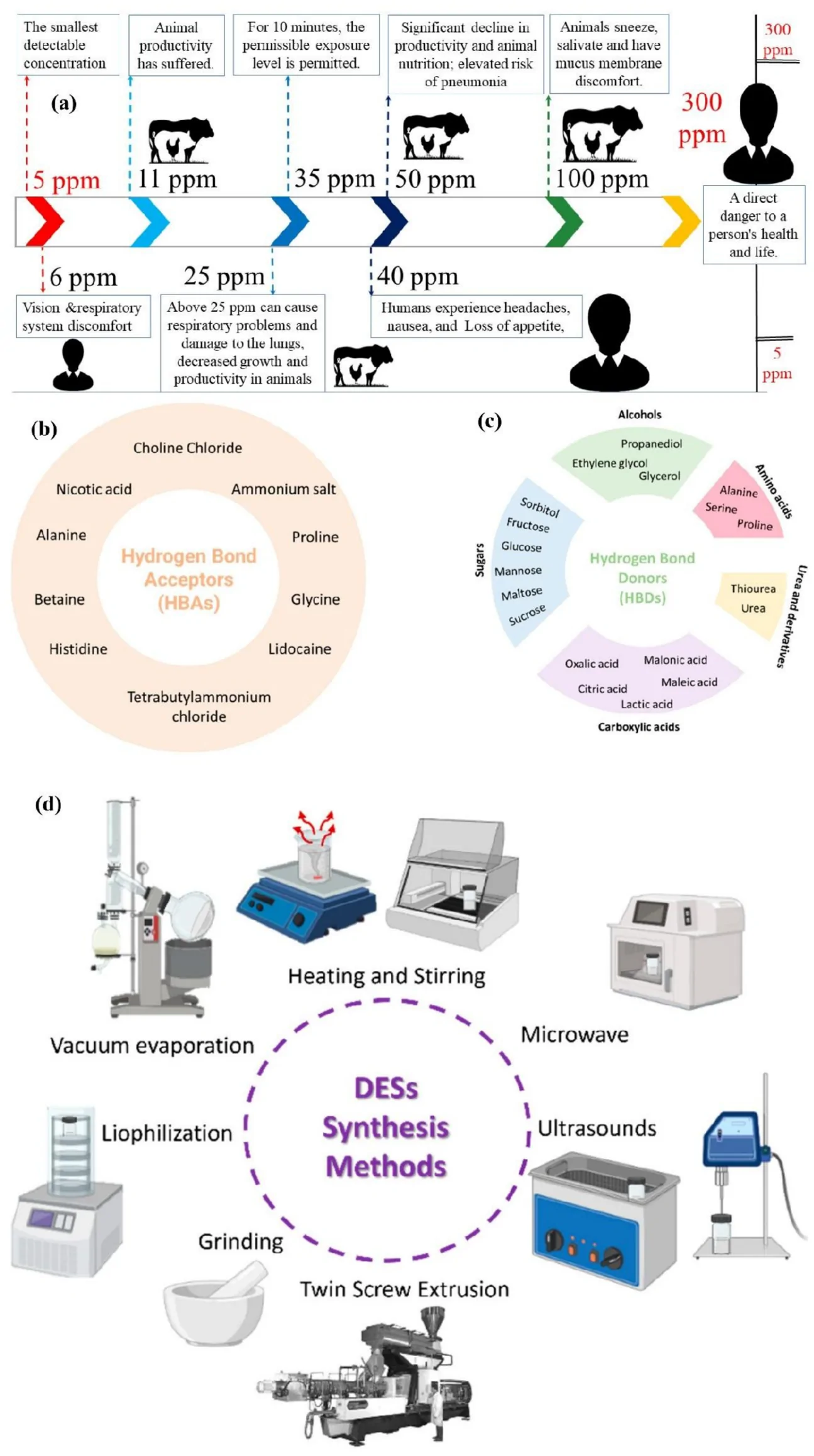
(**a**) Physiological responses of humans and animals to increasing ammonia concentrations. (**b**) Widely used hydrogen-bond acceptors (HBAs) and (**c**) hydrogen-bond donors (HBDs) in the formation of deep eutectic solvents. (**d**) Commonly employed preparation techniques for synthesizing DESs. Reproduced from [31,36], licensed under CC BY 4.0.

A recurring design principle in the reviewed literature (Table 1 and Table 2) is the pairing of a protic, chloride-based HBA with a hydroxyl- or amide-rich HBD, in order to maximize the number of sites available for NH_3_ binding. Ethanolamine hydrochloride (EACl) combined with resorcinol and glycerol in a ternary system exploits both the protic character of the ammonium cation and the multiple hydroxyl groups of glycerol. The resulting EACl/Res/Gly (1:4:5) system reaches an absorption capacity of 0.240 g NH_3_ per g DES at 293 K and 0.1 MPa, with ^1^H NMR analysis indicating that resorcinol’s hydroxyl protons interact most strongly with NH_3_, followed by EACl’s ammonium and hydroxyl protons, and finally the hydroxyl protons of glycerol [4]. Amino-2-propanol hydrochloride, a structurally related protic HBA, has similarly been used to construct DESs with multiple hydrogen-bond sites for NH_3_ capture [14]. A low-viscosity DES formed from guanidine isothiocyanate and acetamide has been applied to both NH_3_ capture and NH_3_/CO_2_ separation, achieving an NH_3_/CO_2_ selectivity of 151 at 303.15 K [5], while NH_4_SCN combined with imidazole produces a “dual active sites” low-viscosity DES for NH_3_ uptake [6]. N-methylacetamide forms DESs with heterocyclic weak acids—imidazole, triazole, and tetrazole—with the tetrazole-based system exhibiting the greatest NH_3_ solubility, consistent with its stronger Brønsted acidity relative to the other two heterocycles [27]. The structural role of the HBA itself has also been examined directly: pairing choline-family chlorides bearing different substituents with ethylene glycol was shown to improve NH_3_ capacity by 5–57% relative to the reline/ethaline benchmark systems, depending on the specific choline derivative used [7]. Urea combined with newly synthesized choline-family salts has similarly been used to construct DESs for efficient ammonia absorption, extending the accessible HBA structural space [26]. More recently, pyridine derivatives have been proposed as a distinct structural class of HBA, expanding the design space beyond conventional quaternary ammonium and imidazolium HBAs specifically for NH_3_ storage (Figure 1) [37,38,39,40,41,42,43,44,45,46,47,48,49].

### 2.2. Multiple/Weak-Acid Sites and Non-Halide DESs

A second design strategy incorporates multiple weak-acid or phenolic functional sites into non-ionic or non-chloride DES frameworks (Table 1 and Table 2), on the premise that distributing interaction sites across the solvent improves both total capacity and low-pressure performance. Non-ionic phenol-based DESs, formed from phenol combined with resorcinol or catechol, were designed with multiple acidic sites and shown to be particularly effective for NH_3_ uptake at low partial pressure [8]. A related ternary system—choline chloride, phenol, and ethylene glycol—exploits the weak Brønsted acidity of phenol, and systematic variations in temperature, pressure, and composition demonstrated highly efficient, reversible NH_3_ absorption in this system [40]. Ethylamine hydrochloride combined with resorcinol and phloroglucinol produces a DES with more than two distinct weak-acid sites, enabling efficient, reversible, and gas-selective NH_3_ uptake [50,51]. To avoid the corrosivity and environmental persistence associated with chloride-containing components, non-chloride DES built from dihydroxybenzoic acids and ethylene glycol have been developed for NH_3_ separation through multiple-site interactions [48], and halogen-free DESs with multiple active sites have similarly been reported [50]. Low-viscosity DESs incorporating multiple weak-acidic groups have also been prepared by pairing a tetramethyl-propanediamine dihydrochloride HBA with phenol, achieving viscosities as low as 48.1 cP and addressing the mass-transfer limitations that high-viscosity DESs typically impose on absorption kinetics [28].

### 2.3. Supramolecular, Metal-Containing, and Hybrid DESs

A third design route incorporates supramolecular hosts or metal centers into the DES structure to introduce binding sites chemically distinct from conventional hydrogen bonding (Table 1 and Table 2). A supramolecular ternary DES combining per-hydroxylated pillar[5]arene (OHP[5]) with EACl and glycerol, EaCl-Gly(1:2)-6%OHP[5], reaches 2.39 mmol/g at 10 kPa and 10.84 mmol/g at 100 kPa (298.2 K), with an NH_3_/CO_2_ selectivity of 84 at 298.2 K that increases to 275 at 313.2 K. Isotherm fitting resolves total uptake into a chemical absorption component (dominant at 0–20 kPa, ΔH ≈ −30.13 kJ/mol) and a physical dissolution component (linear with pressure, 20–100 kPa, ΔH ≈ −12.69 kJ/mol); the system retains 93–95% of its saturation capacity after nine absorption–desorption cycles [9]. An earlier hybrid DES with a flexible hydrogen-bonded supramolecular network—choline chloride, resorcinol, and glycerol—was among the first reported to combine high NH_3_ uptake with good desorption-regeneration performance [24]. Metal chlorides, particularly MgCl_2_, have been incorporated into resorcinol/ethylene-glycol DESs and shown to raise NH_3_ absorption capacity to 0.239 g NH_3_ per g DES at 313 K with good recycling performance, indicating that metal coordination can supplement conventional hydrogen-bonding sites [45]. Because this coordination-based interaction is mechanistically distinct from the two-step hydrogen-bonding/van der Waals pathway described in Section 4 for non-metal DESs, it represents a likely exception to that framework rather than a variant of it. Ternary metal-based DESs with multiple coordinated sites have been developed specifically for NH_3_ storage applications [52], and mesoporous polymers have been used as physical supports for metal-based DESs to enable capture of dilute (low-content) ammonia streams [53,54]. The high viscosity that typically accompanies multi-site and metal-containing formulations has been addressed by encapsulated hybrid DESs, for which an accompanying mass-transfer resistance model clarifies how encapsulation mitigates the diffusion limitations that would otherwise reduce NH_3_ uptake rate [16].

**Table 1:** Deep eutectic solvents designed for NH_3_ capture, separation, and storage (process/application-level studies).

Designed System	Methods	Main Findings
ChCl:urea (1:1.5–1:2.5) [2]	Volumetric solubility measurement, 298.2–353.2 K, 0–300 kPa	NH_3_ solubility rises with pressure, falls with temperature; Henry’s constants derived
ChCl-based DES [3]	Solubility/thermodynamic measurement	Thermodynamic properties of NH_3_ dissolution reported
EACl:Res:Gly (1:4:5) [4]	Absorption vs. temperature/pressure/HBD, spectroscopy	Capacity 0.240 g NH_3_/g DES at 293 K, 0.10 MPa
NH_4_SCN:Imidazole [6]	Absorption vs. temperature/water content/ratio	Low-viscosity “dual active site” DES enables efficient NH_3_ uptake
PhOH:Res or PhOH:Cat (1:3) [8]	Density/viscosity, absorption isotherms, FTIR/NMR/quantum chemistry	Res/PhOH (1:3): 11.862 mol/kg at 101.3 kPa; 6.391 mol/kg at 5 kPa
EaCl-Gly(1:2)-6% OHP[5] [9]	NMR/FTIR characterization	Capacity 10.84 mmol/g; NH_3_/CO_2_ selectivity 275 (313.2 K, 100 kPa)
[Im][SCN]-based DES [10]	Computational (NMR + quantum chemistry)	Hydrogen-bonding origin of NH_3_ absorption mechanistically resolved
ChCl-based DES (generic) [17]	COSMO-SAC + 6 ML models	Physics-informed framework accurately predicts NH_3_ solubility
PIL:EG [20]	Absorption capacity/rate testing	Highest capacity 211 mg NH_3_/g for [Im][NO_3_]/EG (1:3)
ChCl:EG/PD/BD [21]	Density/viscosity, K-K equation, 298.2–353.2 K	Capacities 5.64/11.69 mol/kg at 0.1/1 bar (298.2 K)
Gly + azoles (Tri, Im, Tz, derivatives) [22]	Absorption testing across HBAs	Tri-Gly > Im-Gly; max 0.115 g NH_3_/g DES (Gly-Tri 1:3, 313.15 K)
ChCl:Res:Gly hybrid [24]	NH_3_ uptake/desorption-regeneration cycling	Exceptional capacity + strong regeneration + NH_3_/CO_2_ selectivity
Urea + new choline-family salts [26]	Density/viscosity/solubility measurement	Efficient ammonia absorption reported
MAA + HWAs (imidazole, triazole, tetrazole) [27]	Physical property characterization, isotherms	MAA+tetrazole highest solubility; nonideal isotherm indicates chemical absorption
[bmim][mesylate]:urea (1:1) [30]	Structural (C(2)-H hydrogen bonding) investigation	High NH_3_ absorption identified; C(2)-H is major H-bond contributor
NH_4_SCN + G/EG/U/AT/CL [34]	Absorption/recycling testing	NH_4_SCN-glycerol (2:3): 0.223 g/g at 303 K; stable over 25 cycles
EaCl:urea [39]	Solubility 313.2–353.2 K, 0–300 kPa, K-K correlation	Slightly non-ideal isotherms (chemical character)
GA + PhOH [40]	Absorption rate/capacity, spectroscopy	GA+PhOH (1:1): 6.75/14.72 mol/kg at 10.7/201.0 kPa; equilibrium in 51 s
NH_3_ in reline/ethaline [43]	AIMD simulation	NH_3_-H stabilized by Cl^−^/urea carbonyl O; N stabilized by choline-OH
Tetrazole-based DES [44]	Physicochemical + absorption testing	Multiple H-bond sites give highly efficient uptake
MgCl_2_/Res/EG (0.1:1:2) [45]	Coordination + H-bond absorption testing	Capacity up to 0.239 g/g at 313 K; good recyclability
Halogen-free DES, multiple sites [50]	Absorption testing	Efficient absorption without halide components
EaCl:Res:Phl [51]	Physicochemical + reversibility testing	Multiple weak-acid sites give high, reversible, N_2_/H_2_-selective solubility
ChCl-based DES (generic) [53]	Artificial neural network modeling	NH_3_ solubility linked to T, P, and composition; captures physical vs. chemical trends

**Table 2:** Qualitative comparison of the DES design strategies discussed in Section 2, synthesized across the individual studies cited in each row (added in response to reviewer comment on Section 2).

Design Strategy (Section)	Representative Systems	Absorption Capacity	NH_3_/CO_2_ Selectivity	Viscosity	Cost and Scalability
2.1 Protic, acidic/HBD-rich	EACl/Res/Gly; GI:Acetamide; NH_4_SCN:Imidazole [4,5,6,7,14,26,27]	High (up to 0.244 g/g)	High where reported (up to 151)	Low-moderate; several systems explicitly low-viscosity	Low-cost commodity HBA/HBD; simple one-pot synthesis; good scalability
2.2 Multiple weak-acid/non-halide	Phenol:Res/Cat; ChCl:PhOH:EG; dihydroxybenzoic acid:EG [8,40,48,50]	Moderate-high, notably strong at low partial pressure	Gas-selective in some systems; not systematically compared	Variable; optimized systems reach 48.1 cP [28]	Non-chloride variants reduce corrosivity, but phenolic HBDs raise cost/toxicity; moderate scalability
2.3 Supramolecular, metal-containing, hybrid	OHP[5]-EaCl-Gly; ChCl:Res:Gly; MgCl_2_/Res/EG [9,24,45,52,53,54]	Highest reported (up to 10.84 mmol/g; up to 0.239 g/g)	Highest reported (up to 275)	Generally high; multi-site/metal systems often require encapsulation [16]	Macrocyclic hosts and metal salts increase cost/complexity; scalability more limited
2.4 Azole-based	Gly + triazole/imidazole/tetrazole [22,41,44]	Moderate-high; tracks azole acidity (tetrazole > imidazole/triazole)	Not systematically reported for NH_3_/CO_2_ in this class	Not consistently reported	Azole HBDs costlier than simple alcohols/amides; synthesis remains simple
2.5 IL-derived, hydroxyl-functionalized	[Im][NO_3_]/EG; EaCl:Gly; [bmim][mesylate]:urea [20,29,30]	High (up to 211 mg/g)	Not directly compared in reviewed studies	Often higher than simple binary DES owing to the IL component	IL-derived HBAs are costlier and less scalable than conventional choline-based systems

Note: entries synthesize qualitative trends reported across the individual studies cited in Section 2; no single source in the reviewed literature performed this cross-strategy comparison directly; therefore, capacity, selectivity, and viscosity entries reflect the best-performing or most representative system(s) identified within each design category rather than a controlled head-to-head comparison.

### 2.4. Azole-Based DESs

Azole heterocycles constitute a further HBD class investigated for acid-base and hydrogen-bonding compatibility with NH_3_ (Table 1 and Table 2). Glycerol paired with a series of azole HBDs—triazole, imidazole, tetrazole, and substituted derivatives—forms a family of functional DES for reversible NH_3_ absorption [22]. A subsequent rational-design study extended this azole-based approach through systematic ring substitution to achieve highly efficient, recoverable NH_3_ capture [41]. Consistent with the acidity trend noted in Section 2.1, tetrazole-based DES incorporating multiple hydrogen-bonding sites achieve particularly high NH_3_ uptake, reflecting the stronger acidity of tetrazole relative to imidazole and triazole [44].

### 2.5. Ionic Liquid-Derived and Hydroxyl-Functionalized DESs

**Figure 2:**
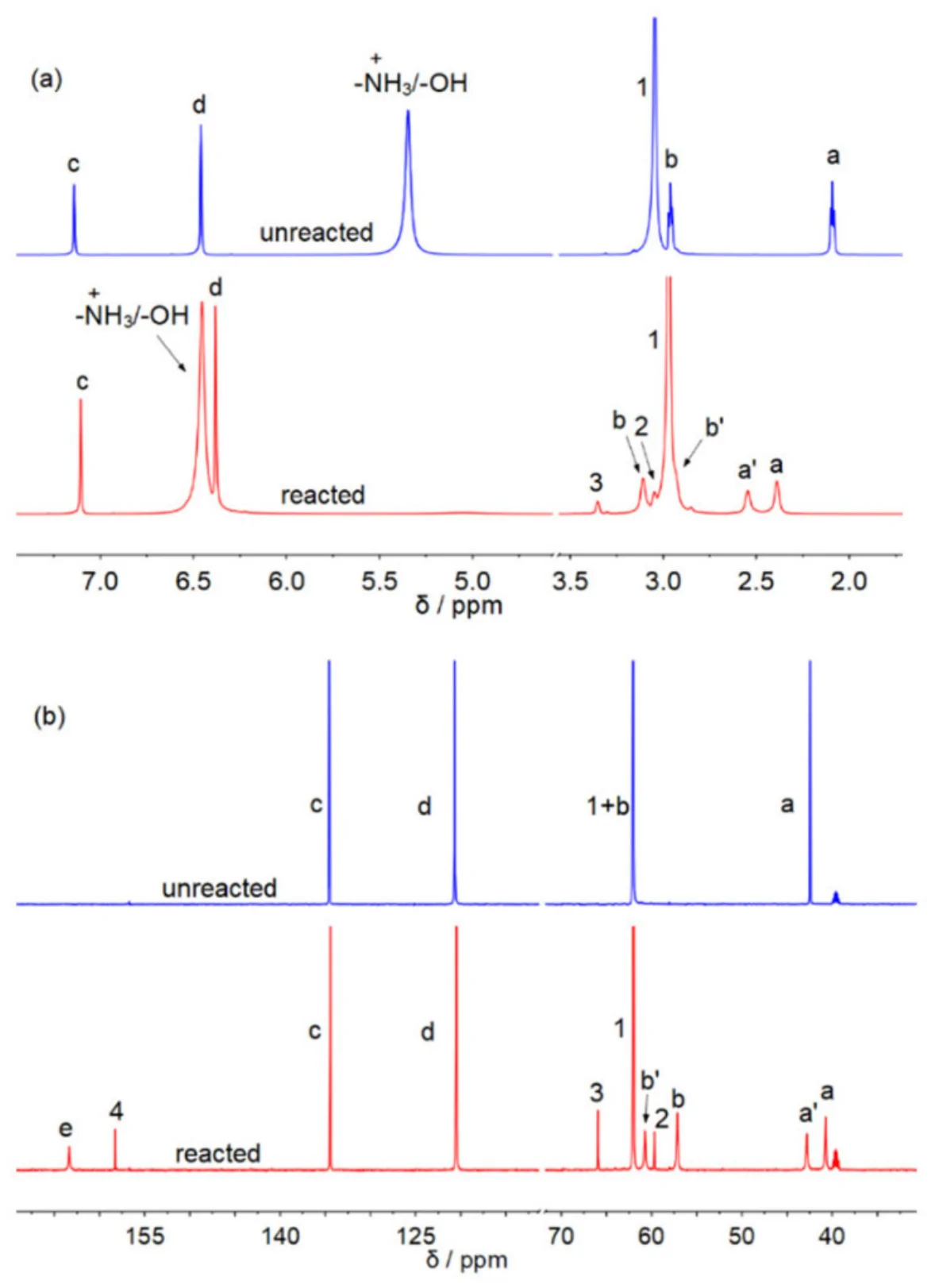
Representative ^1^H NMR spectra of a protic-ionic liquid-derived deep eutectic solvent before and after gas capture, illustrating the chemical-shift evidence typically used to establish hydrogen-bonding interaction between DES and an absorbed gas—the same analytical approach applied to the NH_3_-capture systems discussed in this section. Namely, ^1^H (**a**) and ^13^C (**b**) NMR spectra of [MEAH][Im]-EG (1:3) before and after CO_2_ absorption. Reproduced from [57], licensed under CC BY 4.0.

A final design strategy draws on structure–performance relationships established for ILs (Table 1 and Table 2). IL-based DESs formed by combining a protic IL with ethylene glycol exploit both the acidic protons of the IL and the hydroxyl groups of the glycol for NH_3_ sorption, with [Im][NO_3_]/EG (1:3) achieving the highest reported capacity of 211 mg NH_3_/g among the systems compared [20]. The mechanistic basis for this capacity is consistently attributed to a hydrogen-bonding interaction between the acidic protons of the protic IL component and the sorbed NH_3_ molecule, a claim that is typically substantiated by comparing ^1^H NMR spectra of the DES before and after gas exposure and identifying the resulting chemical-shift changes at the relevant proton sites. Figure 2 illustrates this characterization approach for a structurally related protic-IL-derived deep eutectic solvent, in which new or shifted proton signals appear following gas capture, consistent with the formation of a hydrogen-bonded (or, where amine groups are present, carbamate-type) adduct at the acidic proton site [53,54,55,56,57]. The equivalent NMR evidence reported for the NH_3_-capture systems discussed above [20] follows the same diagnostic logic, though the specific chemical shifts naturally differ for NH_3_ relative to the CO_2_ system shown here.

HBD acidity within DESs has been systematically tuned—described in the source literature as “manufactured” HBD acidity—to balance NH_3_ capture efficiency against reversibility [37]. Ethylamine hydrochloride combined with glycerol has likewise been designed as an NH_3_ absorption medium exploiting the protic ionic character of the HBA [29]. At the mechanistic level, a methanesulfonate-based DES formed from 1-butyl-3-methylimidazolium methanesulfonate and urea was evaluated for ammonia sorption, with structural analysis identifying the imidazolium C(2)-H position as a major contributor to hydrogen bonding with the sorbed gas [30].

## 3. Physicochemical Properties and Solubility Thermodynamics

Beyond capacity optimization, a distinct body of work establishes the dissolution thermodynamics that govern NH_3_ solubility in DESs (Table 3), a prerequisite for rational process design (Table 4). NH_3_ solubility in choline chloride (ChCl) + urea DESs at molar ratios of 1:1.5, 1:2.0, and 1:2.5 was determined by a volumetric method over 298.2–353.2 K and 0–300 kPa. Solubility increases nearly linearly with pressure—indicating physical rather than chemical absorption—and decreases with increasing temperature, consistent with an exothermic dissolution process. Data were correlated with Henry’s law to derive Gibbs free energy, enthalpy, and entropy changes in absorption, which were similar across the three molar ratios (ΔH = −24.05 to −26.22 kJ/mol at 313.2 K), indicating a comparable NH_3_-solvent interaction strength largely independent of exact stoichiometry [2]. A related study quantified NH_3_ solubility and thermodynamics in a broader series of ChCl-based DESs formed with glycerol, ethylene glycol, N-methylurea, and trifluoroacetamide (all 1:2 molar ratio) over 303.15–333.15 K and pressures up to 580 kPa, using an isochoric saturation method. Molality reached as high as 16.27 mol/kg for the ChCl-ethylene glycol system, and Henry’s law-derived thermodynamics confirmed exothermic and spontaneous dissolution (negative ΔH and ΔG) for all four systems, with hydroxyl and amide functional groups identified as the principal NH_3_-trapping sites [3]. Complementary physicochemical characterization determined density, viscosity, and NH_3_ solubility for choline chloride paired with the dihydric alcohols 1,4-butanediol, 2,3-butanediol, and 1,3-propanediol (1:3 and 1:4 molar ratios) over 303.15–333.15 K and pressures up to 420 kPa, with NH_3_/CO_2_ selectivity also evaluated as a function of alcohol structure [21]. Physical properties and NH_3_ solubility were likewise determined for ethylamine hydrochloride + urea DES [39]. At a more predictive level, a theoretical study using the Conductor-like Screening Model for Real Solvents (COSMO-RS) computed physicochemical properties, pairwise interaction energies, and Fukui indices for a series of ammonium salt-based DESs, providing a basis for anticipating HBA/HBD compatibility and DES stability prior to synthesis [36].

## 4. Atomistic and Mechanistic Insights into NH_3_-DES Interactions

Although most reviewed studies address macroscopic absorption performance, a smaller number have probed the fundamental mechanisms driving the NH_3_-DES interaction at the molecular level. Ab initio molecular dynamics (AIMD) simulations of NH_3_ dissolved in reline and ethaline DESs resolved the solvation–shell structure of the gas, identifying which specific components—chloride anions, urea carbonyl oxygen, and choline hydroxyl hydrogen—stabilize NH_3_ at the atomic level [43]. A more extensive computational study combined molecular dynamics simulations with quantum chemistry to investigate NH_3_ absorption in [Im][SCN]-based eutectic solvents, specifically [Im][SCN]-ethylene glycol (1:3) [10]. This work identified a two-stage uptake mechanism: an initial, energetically favored fraction of NH_3_ forms strong hydrogen bonds with the N-H of the imidazolium cation and the hydroxyl group of ethylene glycol (EG), while the majority of subsequently absorbed NH_3_ associates with EG predominantly through van der Waals interactions rather than hydrogen bonding. The calculated interaction energy between NH_3_ and [Im][SCN] was approximately 1.5 times that between NH_3_ and EG, confirming the imidazolium cation as the primary chemical binding site, while NH_3_-NH_3_ hydrogen bonding was found to be negligible, even in saturated DESs. Increasing the temperature from 293.15 K to 333.15 K progressively weakened both van der Waals and Coulombic contributions to the NH_3_-DES interaction; at 323.15 K, the number of hydrogen bonds within the DES-NH_3_ system fell below that of the pure DES, indicating that absorbed NH_3_ spontaneously migrates toward the solvent surface and is released—a molecular-level rationale for the temperature-swing regeneration strategies used throughout the experimental literature. The same framework showed substantially stronger total interaction energy between [Im][SCN]-EG and NH_3_ (−82.61 kJ/mol) than with CO_2_ (−67.83 kJ/mol), offering a mechanistic basis for the NH_3_/CO_2_ selectivity observed experimentally in structurally related DES, and demonstrated that adding 10 wt% water reduces total NH_3_-DES interaction strength from −56.60 to −48.37 kJ/mol, indicating that residual moisture measurably degrades absorption performance [10]. This finding has direct implications for long-term stability under real-world conditions: because many DESs, and NADES in particular, are intrinsically hygroscopic, ambient moisture uptake during storage, transport, and repeated absorption–desorption cycling could progressively weaken the NH_3_-DES interaction in a manner analogous to the deliberately added 10 wt% water modeled here. Reproducibility of laboratory-measured absorption capacities may therefore not translate directly to field conditions unless humidity is controlled or the DES is periodically dried, and systematic studies quantifying performance drift over extended storage and cycling under uncontrolled humidity remain a notable gap in the literature. Beyond these two computational studies, a cooperative acid-base and strong hydrogen-bond interaction mechanism has been proposed to explain highly efficient, selective NH_3_ separation in certain DESs [46], and an N-H hydrogen-bond network structure has been identified as the structural basis for high NH_3_ capacity in a related DES series [47]. Strong proton donors present in glycolic acid-based natural DESs have similarly been examined for their mechanistic contribution to ammonia capture and separation [33].

It is important to note the scope of this two-step (hydrogen bonding followed by van der Waals physical dissolution) mechanism: it has been explicitly established only for a small number of systems—reline/ethaline-type chloride-based DESs [43] and imidazolium-thiocyanate/ethylene-glycol DESs [10]—neither of which contains a coordinating metal center. Whether this framework extends to the azole-based, multiple weak-acid, or supramolecular host–guest systems discussed in Section 2.2, Section 2.3 and Section 2.4 has not been directly verified at the same atomistic level, and should not be assumed given their structurally distinct binding sites. A clear exception is expected for the metal-containing DES discussed in Section 2.3 (e.g., the MgCl_2_-based systems described below [45]), for which direct coordination to the metal center, rather than the sequential hydrogen-bonding/van der Waals pathway, is the more plausible primary interaction. A dedicated computational study directly comparing coordination-driven and hydrogen-bonding-driven NH_3_ uptake within a single metal-containing DES has not yet been reported and would help establish how broadly the two-step picture applies across DES classes.

## 5. Process Modeling, Simulation, and Machine Learning

A further stream of work addresses NH_3_-DES systems at the process and predictive-modeling level, moving beyond single-solvent characterization toward industrial applicability. A process simulation study evaluated DES-based NH_3_/CO_2_ separation applied to melamine production tail gas, a stream that inherently contains both gases and therefore presents a practical separation task [12]. A soft-SAFT equation-of-state model assessed choline chloride-based DESs—formed with ethylene glycol, urea, and glycerol—for selective CO_2_/NH_3_ separation in the same context, predicting absorption isotherms and ideal selectivity directly from the equation of state without exhaustive empirical screening; the ChCl:EG (1:7) formulation gave the best predicted selectivity in a simulated tail gas mixture [32]. At the data-driven end of this spectrum, a physics-informed framework combining COSMO-SAC thermodynamic calculations with machine learning was developed to predict NH_3_ solubility in choline chloride-based DESs, integrating first-principles thermodynamic descriptors with statistical learning [17]. Artificial neural networks have similarly been applied to model NH_3_ absorption capacity in choline chloride-based DESs as a function of composition, temperature, and pressure, offering a purely data-driven complement to the mechanistic and thermodynamic approaches of Section 3 and Section 4 [53].

## 6. NH_3_ Sensing and Analytical Applications

**Figure 3:**
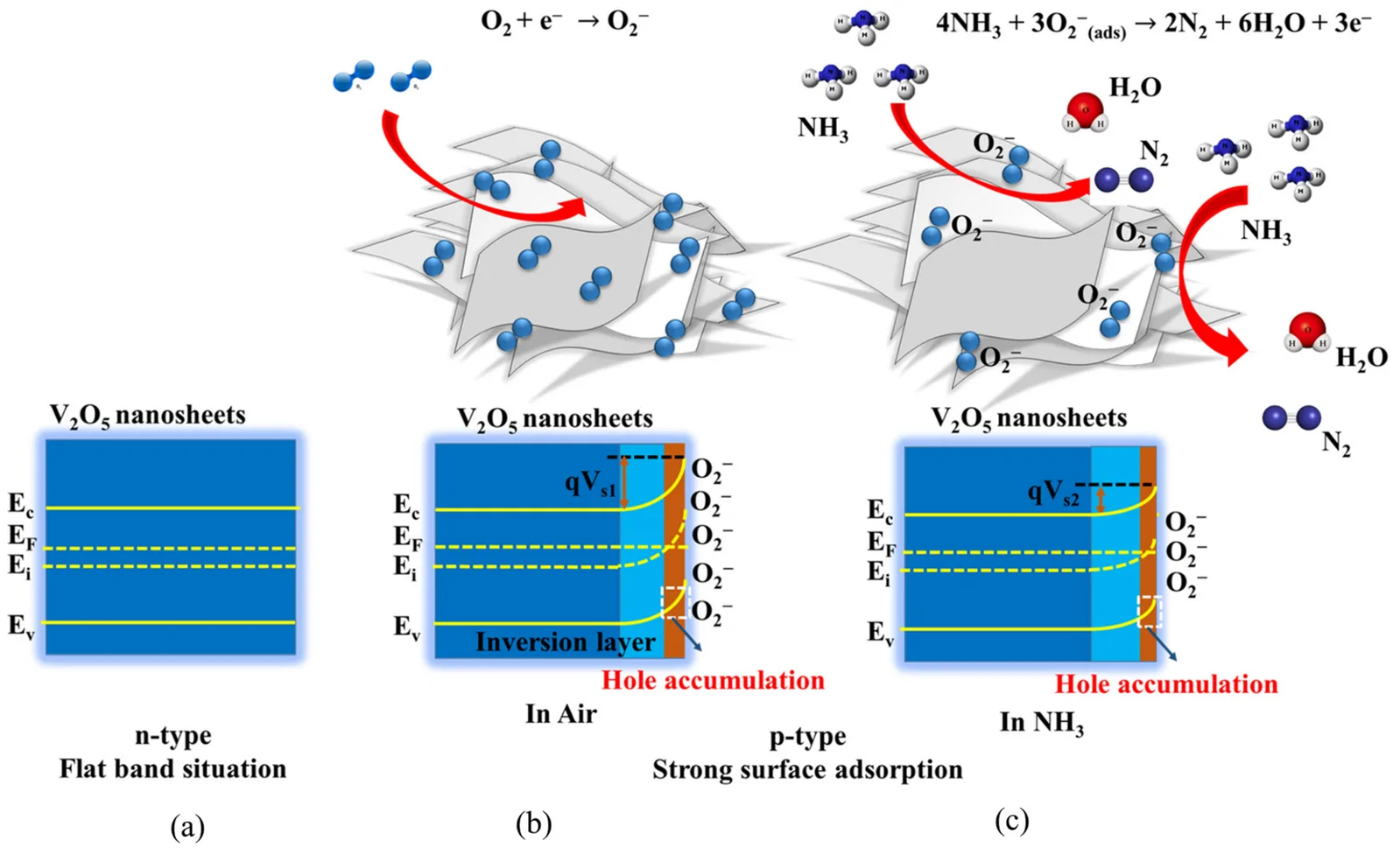
Schematic diagrams of the energy bands of V_2_O_5_ nanosheets (**a**) in vacuum, (**b**) with strong surface oxygen adsorption in air, and (**c**) upon ammonia injection, illustrating a p-type semiconductor NH_3_ sensing mechanism applied to food-spoilage monitoring. Reproduced from [55], licensed under CC BY 4.0.

In contrast to the extensive absorption-capacity literature summarized in Section 2, Section 3, Section 4 and Section 5, comparatively few studies apply DES (Table 5) to the direct sensing or analytical quantification of NH_3_. A NADES prepared from malic acid, xylitol, and water was used to replace pyridine as the catalyst in an air-assisted liquid–liquid microextraction (AALLME) method for gaseous ammonia, coupled with GC–MS derivatization and quantification; the method achieved a limit of quantification of 1.0 µg/L with extraction recoveries of 89–91%, and an AGREEprep greenness score of 0.65, compared with 0.38 for a conventional pyridine/chloroform method and 0.30 for a spectrophotometric indophenol method [1]. A paper-derived, laser-engraved gas sensor consisting of a carbon-based interdigitated electrode array, fabricated by direct laser scribing, incorporated a separate natural DES (lactic acid:glucose:water) to achieve selectivity for NH_3_ over other volatile species; the device was subsequently applied to monitor fish spoilage by detecting NH_3_ evolution [35]. Notably, this NADES sensor does not report the underlying electronic mechanism by which NH_3_ adsorption is transduced into a measurable signal—a gap shared by most DES-sensing studies reviewed here (Section 9). For comparison, food-spoilage-monitoring NH_3_ sensors built on conventional metal-oxide semiconductors have characterized this transduction step in detail: a p-type-like V_2_O_5_ nanosheet sensor applied to the same monitoring context attributes its response to NH_3_-induced band bending at the semiconductor surface, illustrated schematically in Figure 3 [55]. The absence of an equivalent mechanistic diagram for the DES-based sensor in [35] is precisely the kind of connection this review identifies as missing between DES absorption chemistry and DES sensing performance (Section 9).

**Figure 4:**
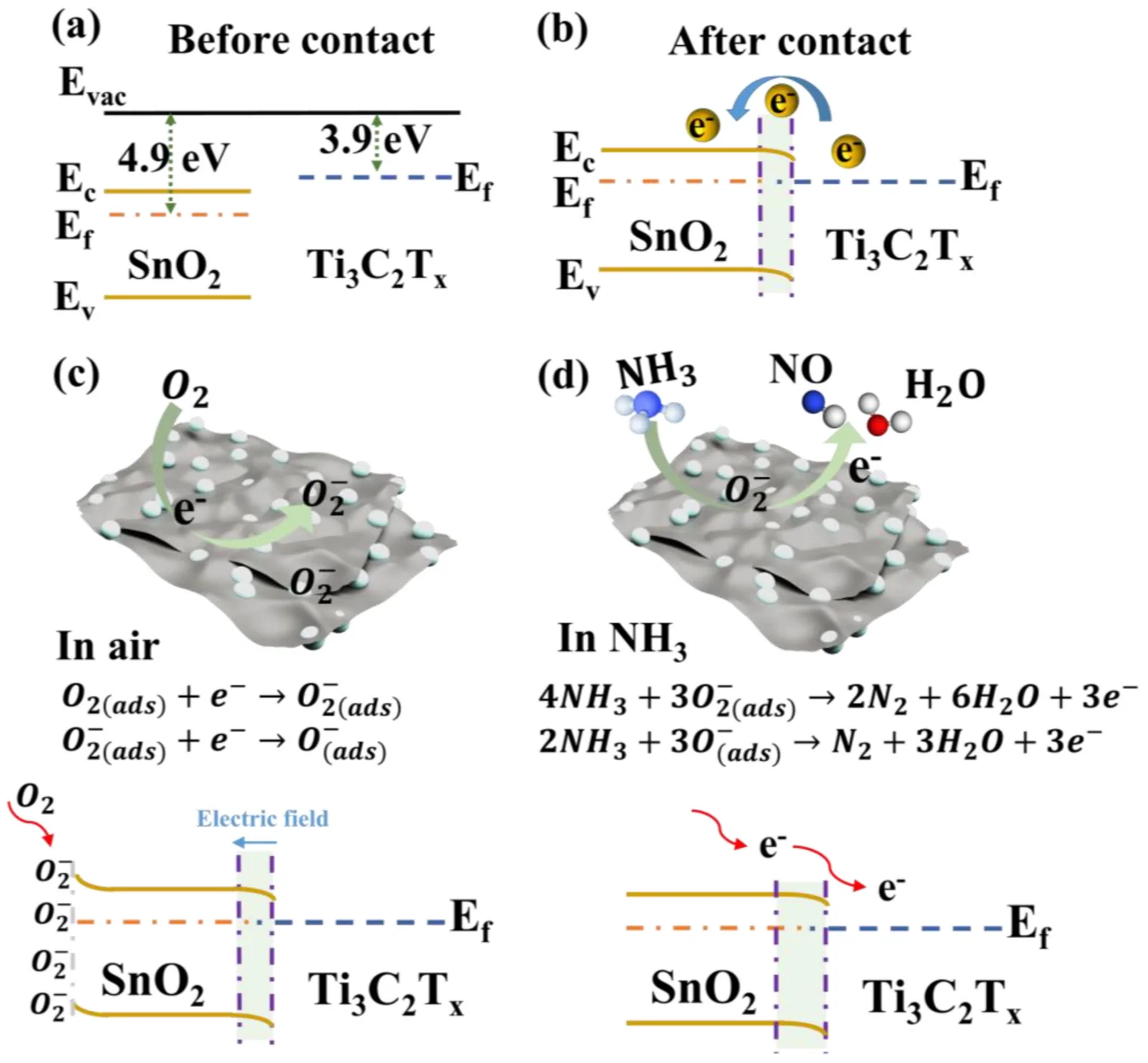
Energy band diagrams of SnO_2_ and Ti_3_C_2_T_x_ (**a**) before and (**b**) after contact, and schematic of the NH_3_ sensing mechanism of the Ti_3_C_2_T_x_/SnO_2_ composite sensor (**c**) in air and (**d**) upon exposure to NH_3_. Reproduced from [56], licensed under CC BY 4.0.

Beyond direct DES-based sensors, DESs have also been used as a synthesis medium to prepare solid-state materials subsequently evaluated for NH_3_ detection. A DES-assisted green synthesis route was used to prepare MAX-phase Cr_2_AlC and its two-dimensional MXene derivative Cr_2_CT_x_, which were evaluated for room-temperature NH_3_ gas detection, with the DES serving as the synthesis medium rather than the sensing element itself [13]. This distinction is important: unlike the NADES-based sensors discussed above, the DES here does not participate in the NH_3_-recognition event—the recognition chemistry instead depends entirely on the resulting MXene surface. To illustrate the class of mechanism such DES-synthesized MXene sensors are expected to exhibit, Figure 4 shows the energy band-based NH_3_ sensing mechanism reported for a structurally related Ti_3_C_2_T_x_ MXene composite sensor, in which surface-adsorbed oxygen depletes charge carriers in air and is displaced upon NH_3_ exposure, producing a measurable signal shift [56,57,58,59,60,61,62,63,64,65].

**Figure 5:**
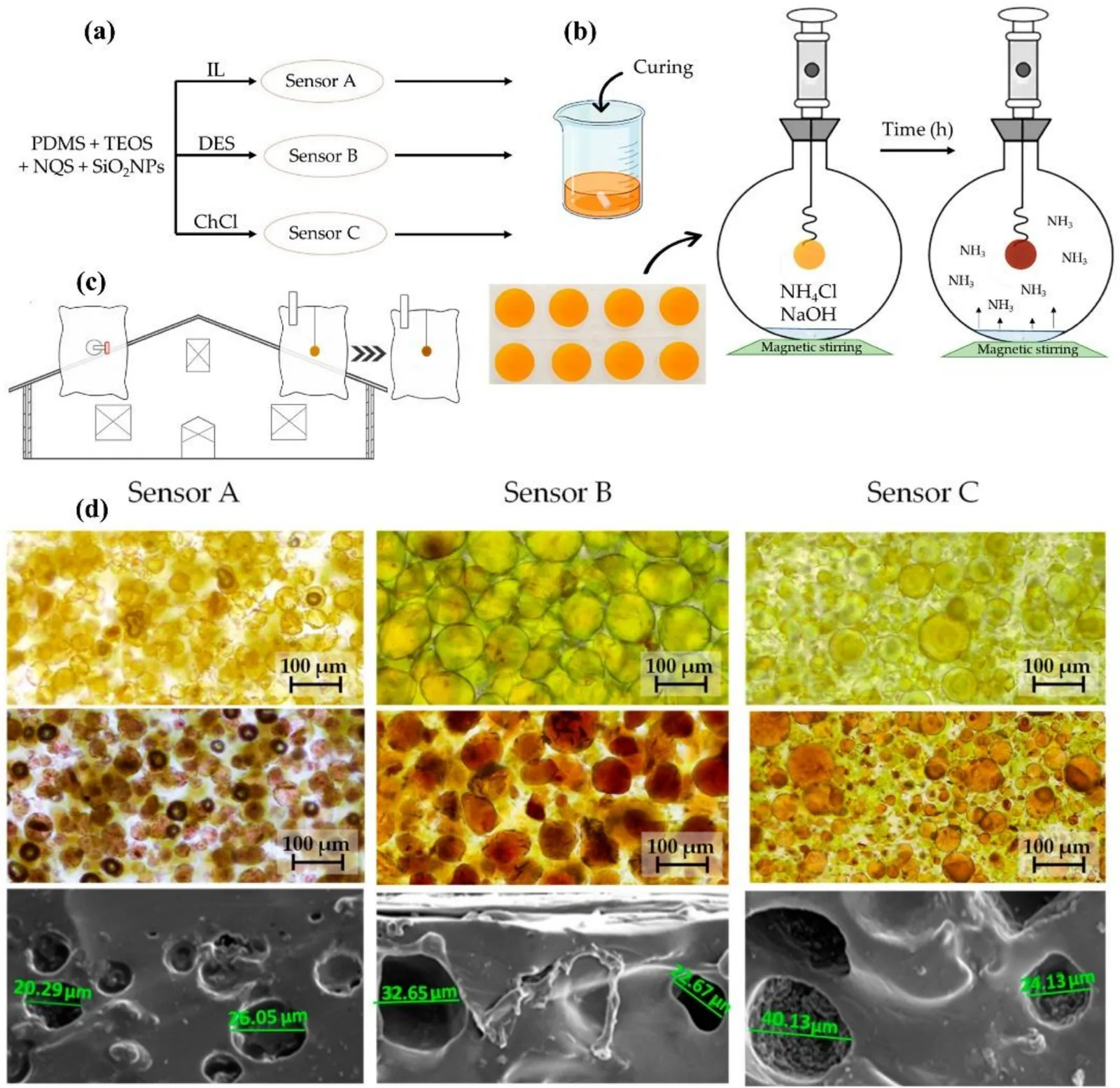
Experimental design and membrane morphology. (**a**) Diagram of the composite material synthesis. (**b**) Setup for generating ammonia gas standards in a 2 L static dilution bottle. (**c**) Field sampling method at the farm, utilizing Tedlar bags and sensor-integrated hermetic bags. (**d**) Optical microscopy (10×) comparing the sensing membranes before and after NH_3_ interaction for Sensors A–C, and corresponding SEM cross-section images revealing the internal structure of each polymer membrane. Reproduced from [64], licensed under CC BY 4.0.

Figure 5 itself is drawn from a separate study unrelated to DES-based co-composting: it illustrates the synthesis, gas-standard generation, and farm field-sampling workflow of a doped PDMS-NQS chemiresistive membrane sensor for ammonia quantification in farm atmospheres, together with the resulting membrane microstructure before and after NH_3_ exposure [64]. This sensor–membrane characterization exemplifies the kind of field-deployed transduction platform discussed in Section 6 and is shown here for that illustrative purpose rather than in connection with the composting study cited immediately above.

At the analytical chemistry end of this application space, a hydrophobic DES composed of tetrapropylammonium bromide and isoamyl alcohol (1:6) was combined with dispersive liquid–liquid microextraction (DLLME) and smartphone-based digital colorimetry to quantify ammonium ion in water samples, likewise achieving recoveries of 89–91% [11].

## 7. Environmental and Electrochemical Applications Beyond Direct Capture

DESs have additionally been applied to NH_3_-related problems outside conventional gas absorption or sensing. Addition of choline chloride-based DESs—formed with either oxalic acid or ethylene glycol—during co-composting of distillery sludge and distiller’s grains waste reduced NH_3_ emissions and associated nitrogen loss during the process, an application of DES chemistry to emission mitigation in a waste-treatment context rather than industrial gas capture [15,66,67,68,69,70,71,72]. In a materials synthesis context, a choline chloride-ethylene glycol DES was used as an electrodeposition medium to prepare a phosphorus-doped CuNi alloy catalyst for selective electrochemical nitrate-to-ammonia reduction. This represents an application of DES chemistry to ammonia production rather than capture, and it is included here to delineate the boundary of the present review’s scope [42].

## 8. Existing Reviews and Positioning of This Work

Several prior reviews address subsets of the literature synthesized above. A critical review addressed DES utilization for reducing the release of hazardous gases—including CO_2_, SO_x_, NO_x_, H_2_S, and NH_3_—to the atmosphere, situating NH_3_ capture within the broader context of DES-based gas remediation [38]. A more focused review compiled recent studies on gaseous ammonia detection and absorption/adsorption technologies, spanning both DES- and metal–organic-framework-based approaches [19]. A further review of ionic liquids and DES for NH_3_ absorption and separation summarized the principal solvent classes discussed in Section 2 of the present work—conventional DES, hydroxyl-functionalized DES, protic DES, multiple-site DES, metallic DES, and DES-supported materials—providing a structural taxonomy of the field [25,70,71,72,73,74,75,76,77,78,79,80,81,82,83,84,85]. None of these prior reviews, however, extends its scope to the DES-based sensing and analytical applications summarized in Section 6, and none draws an explicit connection between the atomistic solvation mechanisms established computationally (Section 4) and the empirical absorption or sensing performance reported experimentally. The present review’s contribution relative to refs. [19,25,38] is therefore threefold: (i) it is, to our knowledge, the first to place DES molecular design (Section 2), thermodynamic and atomistic absorption mechanisms (Section 3 and Section 4), and NH_3_ sensing/analytical applications (Section 6) within a single synthesis; (ii) it explicitly identifies and quantifies the imbalance between these research areas, noting that only a small minority of the studies surveyed address sensing or analytical quantification (Section 9); and (iii) it uses the atomistic hydrogen-bonding/van der Waals framework established for bulk NH_3_ absorption to propose a concrete route for rationalizing and improving the analyte selectivity of DES-based sensors and extraction methods (Section 9.3), a connection not attempted in the prior reviews cited above.

## 9. Research Gaps and Future Perspectives

Synthesis of the 54 articles examined in this review reveals a pronounced imbalance in the current literature. The overwhelming majority of studies address NH_3_ absorption-capacity engineering—systematic variations in HBA/HBD chemistry, molar ratio, and physical properties to maximize uptake and selectivity (Section 2)—together with the solubility thermodynamics required to characterize this performance (Section 3). By contrast, only four of the reviewed articles describe an NH_3_ sensing device or analytical quantification method [1,11,13,35], and none of these four explicitly connects its sensing or extraction selectivity mechanism to the atomistic-level solvation chemistry established computationally in Section 4 [10,43]. The mechanistic studies that do exist—resolving hydrogen-bonding and van der Waals contributions to NH_3_ dissolution, and identifying the temperature-dependent reversal of hydrogen-bond populations that underlies regeneration—were developed exclusively in the context of bulk gas absorption rather than sensing transduction [86,87,88,89,90,91,92]. This represents a clear opportunity: the same interaction-energy and hydrogen-bond-competition frameworks that explain NH_3_/CO_2_ selectivity in absorption applications [10] could, in principle, be used to rationalize and improve the analyte selectivity of DES-based sensors and extraction methods, but this connection has not yet been made explicit in the literature surveyed here [93,94,95,96,97,98,99,100].

### 9.1. Structure–Performance Relationships in DES Design

The design strategies cataloged in Section 2 collectively demonstrate that NH_3_ absorption capacity in DES is governed by a hierarchy of molecular factors, with hydrogen-bond donor (HBD) acidity emerging as the primary determinant, followed by the density of interaction sites and the physical properties (particularly viscosity) that influence mass transfer. Among the various DES families examined, systems incorporating protic ammonium salts with multiple hydroxyl- or amide-rich HBDs consistently achieve the highest gravimetric capacities—up to 0.244 g NH_3_/g DES for EaCl:glycerol [14] and 0.240 g NH_3_/g DES for EACl:Res:Gly (1:4:5) [4]—demonstrating that the combination of strong Brønsted acidity and multiple hydrogen-bonding sites is the most effective design principle currently available.

This empirical observation aligns with the broader understanding of DES structure–function relationships established in the literature. Smith et al. [89] and Florindo et al. [91] emphasized that the physicochemical properties of DESs—including their ability to interact with target molecules—derive primarily from the nature and strength of hydrogen bonding between the HBA and HBD components, as well as the resulting supramolecular network structure. The systematic tuning of HBD acidity described by Jiang et al. [37] represents a rational approach to optimizing the balance between absorption capacity and reversibility, a trade-off that is fundamental to practical NH_3_ capture applications. Importantly, this balance is not merely a function of acidity strength but also involves the cooperative effects of multiple weak-acid sites, as demonstrated by the high capacities achieved by phenol-based [8] and azole-based [22,41] systems despite their comparatively moderate individual acidity.

The incorporation of supramolecular hosts such as pillar[5]arene [9], and metal centers [45,52] represents a further evolution in DES design, introducing binding sites chemically distinct from conventional hydrogen bonding. The exceptional NH_3_/CO_2_ selectivity achieved by the EaCl-Gly(1:2)-6%OHP[5] system (275 at 313.2 K) [9] suggests that supramolecular confinement and pre-organization of binding sites can enhance selectivity beyond what is achievable through simple hydrogen-bonding interactions alone. This principle finds parallels in the broader DES literature, where structure–function relationships have been extensively explored for applications ranging from lignin extraction [88] to biomass pretreatment [87] and food contaminant extraction [92,95].

### 9.2. Atomistic Mechanisms: From Bulk Absorption to Interfacial Phenomena

The mechanistic studies reviewed in Section 4, particularly the AIMD simulations of Malik and Kashyap [43] and the comprehensive computational investigation by Cao et al. [10], provide atomistic-level resolution of the NH_3_-DES interaction. These studies establish a two-stage mechanism: an initial, energetically favored chemical absorption phase involving specific hydrogen bonding at donor sites (N-H of imidazolium cation, hydroxyl groups of EG, chloride anions, and urea carbonyl oxygen), followed by a physical dissolution phase dominated by van der Waals interactions once primary sites are saturated. The calculated interaction energies—with NH_3_-[Im][SCN] approximately 1.5 times stronger than NH_3_-EG interactions [10]—quantitatively rationalize the experimentally observed absorption capacities and selectivities.

Critically, this mechanistic framework can be extended beyond bulk absorption to understand DES behavior at interfaces, a consideration that is directly relevant to both gas-sensing and analytical extraction applications. Bexeitova et al. [59] demonstrated through computational studies that DES components exhibit distinct interfacial behavior on polymer surfaces. Specifically, DES-mediated oil–water interfacial behavior significantly influences the distribution and transport of solutes. This interfacial perspective is essential for understanding DES-based sensing platforms, where the detection event occurs at the interface between the DES phase and the gas or liquid analyte rather than in the bulk solvent. Similarly, the molecular interactions between polyaniline functional groups and NH_3_, elucidated by Nulimu et al. [60] through theoretical calculations, provide a framework for understanding how conducting polymers—often used in conjunction with DESs in sensor platforms—contribute to analyte recognition and signal transduction.

The temperature-dependent reversal of hydrogen-bond populations observed in AIMD simulations [10]—where the number of hydrogen bonds within the DES-NH_3_ system falls below that of pure DES at elevated temperatures (323.15 K)—provides a molecular rationale for temperature-swing regeneration. This mechanistic understanding is essential for process design, as it establishes the thermodynamic driving force for desorption and enables optimization of regeneration conditions. The COSMO-RS-based theoretical studies of ammonium-salt-based DESs and the physics-informed machine learning framework combining COSMO-SAC with ML [17] represent important steps toward predictive design, potentially reducing the need for exhaustive experimental screening. The application of artificial neural networks to model NH_3_ absorption capacity [53] further demonstrates the potential of data-driven approaches to accelerate DES discovery.

### 9.3. Bridging the Gap: From Absorption Chemistry to Sensing Performance

The most striking imbalance revealed by this review is the pronounced disconnect between the extensive absorption-capacity literature (Section 2, Section 3, Section 4 and Section 5) and the comparatively sparse sensing/analytical literature (Section 6). While 50 of the 54 core reviewed articles address NH_3_ absorption, separation, or related process applications, only four describe NH_3_ sensing or analytical quantification [1,11,13,35], and none of these four explicitly connects its sensing or extraction selectivity mechanism to the atomistic-level solvation chemistry established computationally [10,43].

This gap represents both a limitation of the current literature and a significant opportunity for future research. The same interaction-energy and hydrogen-bond-competition frameworks that explain NH_3_/CO_2_ selectivity in absorption applications could, in principle, be used to rationalize and improve the analyte selectivity of DES-based sensors and extraction methods. For example, the sensing mechanism of the lactic acid:glucose:water NADES-based sensor described by Reynolds et al. [35]—which achieved selective NH_3_ detection with a limit of detection of 0.33% and was applied to fish spoilage monitoring—has not been characterized at the molecular level. Yet the atomistic understanding of NADES solvation chemistry, including the role of specific functional groups in hydrogen bonding with NH_3_, could potentially explain the observed selectivity and guide further optimization.

The broader sensor literature offers established frameworks for understanding such transduction mechanisms. For instance, the energy-band-based mechanisms elucidated for semiconductor NH_3_ sensors [55,56]—where surface-adsorbed oxygen depletes charge carriers in air and is displaced upon NH_3_ exposure—provide a template for how chemiresistive transduction could be integrated with DES-based recognition. Studies of polyaniline-based NH_3_ sensors [66,67] have demonstrated how protonation–deprotonation processes can be transduced through changes in electrical conductivity, while chemiresistive sensors based on organic materials [68] have established strategies for enhancing sensitivity through controlled doping and morphology engineering. The work of Yang et al. [69] on bacterial cellulose/polyaniline sensors and Mikhaylov et al. [70] on TiO_2_/PANI-DBSA nanocomposites further illustrates how the interplay between organic recognition elements and inorganic supports can be optimized for NH_3_ detection. Similarly, the modulation of Fe^2+^/Fe^3+^ ratios and oxygen vacancies in barium hexaferrite sensors [71], the spin crossover phenomenon in metal complexes [72], and the design principles for metal–organic framework sensors [75,77,78,79] offer mechanistic frameworks that could be adapted for DES-based sensing platforms.

The analytical extraction applications of DES, particularly the air-assisted liquid–liquid microextraction (AALLME) method catalyzed by NADES [1] and the dispersive liquid–liquid microextraction (DLLME) combined with smartphone colorimetry [11], represent another domain where deeper mechanistic understanding would be beneficial. The AGREEprep greenness score of 0.65, achieved by the NADES-based method [1]—compared with 0.38 for conventional pyridine/chloroform and 0.30 for spectrophotometric indophenol methods—demonstrates the green analytical potential of DES, but the molecular basis for extraction selectivity remains to be elucidated. Recent advances in DES-integrated sensors for pharmaceutical sensing [73] and the broader application of natural deep eutectic solvents as extraction media [98] highlight the growing recognition of DES as sustainable alternatives in analytical chemistry, yet the mechanistic underpinnings of these applications remain underdeveloped.

### 9.4. Comparative Context: DESs vs. Ionic Liquids and Other Absorbents

The performance of DESs for NH_3_ capture compares favorably with that of ionic liquids (ILs) on multiple metrics, but this comparison merits critical examination. While several DES systems report NH_3_ absorption capacities exceeding those of many functionalized ILs [4,9,18,20,21,22], this comparison is not always straightforward due to differences in measurement conditions (temperature, pressure) and reporting units (gravimetric vs. molar). The highest reported DES capacity—0.244 g NH_3_/g DES [14]—approaches the theoretical maximum for chemisorption-based systems, suggesting that the design space for further capacity improvement is becoming constrained.

However, DESs offer advantages beyond raw capacity. The simplicity and economy of DES preparation, by direct mixing of HBA and HBD without complex ionic synthesis, represent a significant practical advantage over ILs for large-scale applications [4,10]. This advantage is not unconditional, however: many DESs—particularly the multiple weak-acid-site, phenolic, and metal-containing formulations discussed in Section 2.2 and Section 2.3—exhibit substantially higher viscosity than typical functionalized ILs, which directly impairs mass-transfer rates, raises pumping energy requirements, and can limit achievable cycle times in continuous absorption–desorption processes. Viscosities as low as 48.1 cP have been achieved only through deliberate low-viscosity design [28], underscoring that low viscosity is an active engineering target rather than an inherent DES property. A fair comparison with ILs should therefore weigh the synthetic simplicity and cost advantages of DES against this viscosity-related mass-transfer penalty rather than treating ease of synthesis alone as decisive. The biocompatibility and biodegradability of many DESs, particularly NADES, further distinguish them from conventional ILs and organic solvents [89,91]. Toxicity profiling of ammonium-based DESs [31,94] has provided important safety-relevant data, and the emerging understanding of DES cytotoxicity [99] is essential for applications involving biological or environmental exposure. The tunability of DES composition—extending beyond simple binary systems to ternary, supramolecular, and metal-containing formulations—offers a wider design space than is typically accessible with ILs [9,45,52]. These sustainability and cost credentials, however, are better assessed on a system-by-system basis than assumed by default for DESs as a class: HBA/HBD components range from inexpensive, biodegradable natural metabolites (sugars, amino acids, organic acids) to costlier synthetic macrocyclic hosts or metal salts (Section 2.3), and biocompatibility, toxicity, and environmental persistence vary correspondingly with composition rather than being an intrinsic property of the DES concept as a whole [94,99].

The process-scale modeling and simulation studies reviewed in Section 5 [12,32] provide a bridge between fundamental understanding and industrial application. The soft-SAFT equation-of-state modeling of ChCl:EG (1:7) for NH_3_/CO_2_ separation [32] and the process simulation of DES-based melamine tail gas treatment [12] demonstrate that the thermodynamic frameworks developed for ILs and conventional solvents can be adapted to DES. The physics-informed machine learning approach combining COSMO-SAC with ML [17] represents a particularly promising direction for accelerating the design of DESs for specific separation tasks.

### 9.5. Integrating Experimental and Computational Approaches

The integration of experimental and computational methods has been essential for advancing the understanding of NH_3_-DES interactions. The computational studies by Cao et al. [10] and Malik and Kashyap [43] have provided atomistic-level insights that complement experimental characterization (NMR, FTIR, absorption isotherms), establishing a consistent mechanistic picture. This integrated approach aligns with the broader trend in DES research, where computational methods ranging from DFT [74,82] to molecular dynamics [59] and machine learning [17,53] are increasingly used to complement experimental studies. The atomistic investigation of polystyrene supported by amidinium-based ILs for CO_2_ absorption [58] demonstrates the level of mechanistic detail that can be achieved for polymer-supported solvent systems, providing a methodological template for studying supported DES systems for gas capture. The theoretical study of Nulimu et al. [60] on polyaniline functional groups for NH_3_ sensing similarly illustrates how computational chemistry can reveal the specific atomic-level interactions responsible for analyte recognition, offering a pathway to bridge the gap between absorption chemistry and sensing performance.

The investigation of DES-assisted oil–water interfacial behavior on polystyrene surfaces by Bexeitova et al. [59] is particularly relevant to sensing and extraction applications, as it demonstrates that the presence of DES can significantly alter interfacial properties and solute distribution. This interfacial perspective is essential for understanding DES-based sensors, where the recognition event occurs at the interface between the DES phase and the gas or liquid analyte. The broader computational literature on gas capture and sensing [60,61,62,63,64,74,80,81,82,100] provides methodological frameworks and mechanistic insights that can be adapted to DES-based systems.

### 9.6. Outlook and Future Directions

The synthesis presented in this review identifies several clear directions for future research:

1. Mechanistic characterization of DES-based sensors: The most urgent need is to establish the molecular mechanisms by which NH_3_ adsorption in DESs is transduced into a measurable analytical signal in the few DES-based sensors reported to date [1,11,13,35]. This requires coupling spectroscopic characterization (e.g., in situ Raman or FTIR) with computational modeling (DFT or MD) of the DES–analyte interaction at the sensing interface, following the methodological approaches demonstrated by Nulimu et al. [60] for polyaniline systems.

2. Bridging absorption and sensing design: The atomistic frameworks developed for bulk absorption—particularly the two-stage hydrogen-bonding/van der Waals mechanism [10,43]—should be systematically applied to understand and predict sensing selectivity. This requires developing structure–performance relationships for DES-based sensors analogous to those established for absorption.

3. Integration of sensing platforms: The combination of DESs with established sensing platforms—chemiresistive polymers [66,67,68,69], metal oxides [55,71,84], carbon nanomaterials [67,85,86], and metal–organic frameworks [75,77,78,79]—offers opportunities to exploit both the recognition specificity of DESs and the transduction efficiency of conventional materials. The recent work on MXenes synthesized via DES-assisted routes [13] and the broader application of DESs in materials synthesis [42] suggest promising directions for hybrid sensor development. Realizing this integration will also require addressing potential compatibility issues between DESs and the materials of established sensor platforms: many DESs are mildly acidic or hygroscopic and could dissolve, swell, or plasticize the polymer matrices used in chemiresistive and optical sensors, or promote corrosion or degradation of metal-oxide or metallic electrode surfaces over repeated exposure cycles. These interfacial-compatibility and long-term-stability questions have not yet been systematically examined for DES-sensor hybrids and represent a necessary complement to the mechanistic and selectivity work outlined above.

4. Process-scale validation: The process modeling and simulation studies reviewed in Section 5 should be extended to validate the predicted performance of DES-based NH_3_ capture processes at pilot scale. This requires developing robust thermodynamic models for NH_3_-DES systems that account for the complex, non-ideal behavior observed experimentally [2,3,21,39].

5. Life-cycle and sustainability assessment: While DESs are generally regarded as green solvents, comprehensive life-cycle assessments of DES-based NH_3_ capture and sensing technologies are needed to quantify their environmental benefits relative to conventional approaches [15,31]. The application of DESs to emission mitigation in waste-treatment contexts [15] demonstrates that the sustainability benefits of DESs extend beyond direct capture applications.

6. Exploration of understudied DES classes: The design space for NH_3_-capture DESs is far from exhausted. Recently proposed classes, including pyridine derivatives as HBAs [49], non-halide DES [48,50], and ternary metal-based systems [52], warrant further investigation, as do the combination of DESs with mesoporous supports for dilute ammonia capture [54] and encapsulation strategies to overcome viscosity barriers [16].

7. Expanding analytical applications: The promising results of DES-based AALLME [1] and DLLME [11] for ammonia quantification should be extended to other analytical applications, including real-time environmental monitoring, industrial process control, and biomedical diagnostics. The recent review by Karibayev et al. [65] on DESs in analytical extraction of carcinogens provides a methodological framework for developing such applications.

## 10. Conclusions

This critical review has systematically examined deep eutectic solvent research for ammonia capture, detection, and analytical applications, revealing substantial progress alongside significant gaps that define the frontier of this field.

Molecular design strategies for NH_3_-capturing DESs have advanced considerably, yielding systems with absorption capacities approaching 0.244 g NH_3_ per gram of DES, NH_3_/CO_2_ selectivities exceeding 275, and viscosities as low as 48.1 cP. The expansion from simple choline chloride–urea systems to sophisticated architectures incorporating protic salts, multiple weak-acid sites, azole heterocycles, supramolecular hosts, and metal centers demonstrates that DESs are genuinely designable solvents with rationally optimizable structure–performance relationships.

Atomistic understanding has progressed through complementary computational and experimental approaches, resolving a two-stage absorption mechanism: initial chemical absorption via hydrogen bonding at specific donor sites, followed by physical dissolution governed by van der Waals interactions. This mechanistic framework, combined with process modeling and machine learning, is beginning to enable predictive design rather than empirical screening.

However, the review reveals a pronounced imbalance. While the overwhelming majority of studies address absorption-capacity engineering, only four articles describe NH_3_ sensing or analytical quantification. Critically, none of these sensing studies explicitly connect their detection mechanisms to the atomistic solvation chemistry established for bulk absorption. This disconnect represents the most significant gap in the current literature.

Looking forward, several priorities emerge: mechanistic characterization of DES-based sensors, systematic application of atomistic frameworks to understand sensing selectivity, integration of DESs with established sensing platforms, process-scale validation, life-cycle assessments, and an extension of DES-based analytical methods to real-time monitoring applications. Bridging the gaps between absorption chemistry and sensing transduction, molecular mechanism and device performance, and laboratory discovery and industrial application will define the next phase of DES research for ammonia-related technologies.

## Figures and Tables

**Table 3:** Reported NH_3_ absorption capacities from Table 1 and Table 2 and Section 2 and Section 3, converted to a single unit system (mmol NH_3_ per g DES) using M(NH_3_) = 17.03 g/mol, with the original reported value and measurement conditions retained for traceability (added in response to reviewer comment on data comparability). “n.r.” = not reported in the cited source.

System [Ref]	Reported Value (Original Units)	Converted Capacity (mmol NH_3_/g DES)	T (K)	P (kPa)	Notes
EACl:Res:Gly (1:4:5) [4]	0.240 g/g	14.09	293	100	
AMPH-Cl:Glycerol (2:1) [14]	0.244 g/g	14.33	293	100 (1 bar)	Highest gravimetric capacity reported in this review
Imidazole:Resorcinol (1:1) [18]	0.238 g/g	13.98	293.15	100	
PhOH:Res (1:3) [8]	11.862 mol/kg	11.86	n.r.	101.3	Same system at 5 kPa: 6.391 mmol/g
EaCl-Gly(1:2)-6%OHP[5] [9]	10.84 mmol/g	10.84	298.2	100	Same system at 10 kPa: 2.39 mmol/g
[Im][NO_3_]/EG (1:3) [20]	211 mg/g	12.39	n.r.	n.r.	Conditions not reported in source
ChCl:EG/PD/BD [21]	11.69 mol/kg	11.69	298.2	100 (1 bar)	Same system at 0.1 bar: 5.64 mmol/g
Gly-Tri (1:3) [22]	0.115 g/g	6.75	313.15	n.r.	Highest of the azole-HBD series screened
[TMPDA]Cl_2_:PhOH [28]	4.49 mol/kg	4.49	298.2	13.3	Viscosity as low as 48.1 cP
EaCl:Glycerol [29]	9.631 mol/kg	9.63	298.2	106.7	
NH_4_SCN-glycerol (2:3) [34]	0.223 g/g	13.09	303	n.r.	Stable over 25 absorption–desorption cycles
GA+PhOH (1:1) [40]	14.72 mol/kg	14.72	n.r.	201.0	Same system at 10.7 kPa: 6.75 mmol/g
TrZ+Tri (1:2) [47]	0.241 g/g	14.15	298.15	n.r.	
2-Aminopyridine:Resorcinol [49]	0.185 g/g	10.86	303.15	n.r.	NH3/CO2 selectivity 92.2
Ternary metal-based DES [52]	10.02 mol/kg	10.02	313.2	6.4	
MgCl_2_/Res/EG (0.1:1:2) [45]	0.239 g/g	14.03	313	n.r.	Metal-coordination-supplemented uptake
ChCl:EG (highest of series) [3]	16.27 mol/kg	16.27	303.15–333.15	up to 580	Maximum molality across a 4-system series; single peak value, not isothermal

Note: mol/kg and mmol/g are numerically identical and are reported here without conversion. Because the underlying studies used different experimental methods (volumetric, gravimetric, isochoric saturation) and, in several cases, did not report both temperature and pressure, the values above should be compared with caution and are intended to aid orientation across studies rather than to support precise ranking of solvent performance.

**Table 4:** Deep eutectic solvents designed for NH_3_ absorption (fundamental solvent–gas interaction studies).

Designed System	Methods	Main Findings
GI:Acetamide (1:2) [5]	Solubility/selectivity, Henry’s law correlation	NH_3_/CO_2_ selectivity 151 at 303.15 K; reversible over 5 cycles
EG + choline-family chlorides (1:2) [7]	Density/viscosity/refractive index, solubility	HBA structure change improves NH_3_ capacity 5–57% vs. ethaline
DES-0/DES-EN processes [12]	Aspen process simulation, thermodynamic modeling	DES-based NH_3_/CO_2_ separation modeled for melamine tail gas recovery
AMPH-Cl:Glycerol (2:1) [14]	Absorption testing	Capacity 0.244 g/g at 293 K, 1 bar; abundant H-bond network
ChCl/oxalic acid and ChCl/EG added to compost [15]	34-day co-composting trial	NH_3_ emissions cut 73%, N loss cut 54% (CE treatment)
Encapsulated hybrid DES [16]	Mass-transfer resistance modeling	Encapsulation overcomes viscosity barrier limiting NH_3_ capture
Imidazole:Resorcinol (1:1) [18]	Absorption capacity, NMR/FTIR/quantum chemistry	Capacity 0.238 g/g at 293.15 K, 0.1 MPa
Review: sensors + DES/MOF absorption [19]	Literature review	Compiles chemoresistive sensors and DES/MOF NH_3_ capture approaches
Review: DES for toxic gas capture [23]	Literature review	Summarizes efficient, reversible SO_2_/NH_3_/H_2_S/NO_2_/NO capture by DES
Review: ILs and DES for NH_3_ [25]	Literature review	Synthesizes composition-performance links across DES/IL classes
[TMPDA]Cl_2_:PhOH (1:3–7) [28]	Viscosity/absorption testing	Viscosity as low as 48.1 cP; 4.49 mol/kg at 298.2 K, 13.3 kPa
EaCl:Glycerol [29]	Absorption capacity testing	Capacity 9.631 mol/kg at 298.2 K, 106.7 kPa
ChCl:EG/UR/GL [32]	soft-SAFT equation-of-state modeling	ChCl:EG (1:7) gives best NH_3_/CO_2_ selectivity in simulated tail gas
GA + XYL NADES [33]	Absorption/spectral/quantum chemical analysis	High-efficiency reversible NH_3_ capture demonstrated
DES with tuned HBD acidity [37]	Systematic acidity–absorption study	HBD acidity tuned to balance uptake efficiency and reversibility
Review: DES for hazardous gas mitigation [38]	Critical review	DES positioned as tunable, low-cost alternative for CO_2_/SO_x_/NO_x_/H_2_S/NH_3_ capture
Azole-based DES (rational design) [41]	Design/absorption testing	Highly efficient, reversible capture via rational azole selection
Cooperative acid-base + H-bond DES [46]	Selectivity/separation testing	High selectivity via combined acid-base and strong H-bond interaction
N-H hydrogen-bond network DES [47]	Absorption/selectivity, spectral+theoretical	TrZ+Tri (1:2): 0.241 g/g at 298.15 K with multi-gas selectivity
Dihydroxybenzoic acids + EG (non-chloride) [48]	Multiple-site separation testing	Effective NH_3_ separation without chloride components
2-Aminopyridine:Resorcinol [49]	Thermal stability, NMR/FTIR, absorption testing	Capacity 0.185 g/g at 303.15 K; NH_3_/CO_2_ selectivity 92.2
Ternary metal-based DES [52]	Multi-site design/absorption testing	NH_3_ solubility 10.02 mol/kg at 313.2 K, 6.4 kPa
Mesoporous-polymer-supported metal-based DES [54]	Support integration + absorption testing	Enhanced capture of low-content (dilute) ammonia

**Table 5:** Deep eutectic solvents applied to NH_3_ detection and analytical determination.

Designed System	Methods	Main Findings
NADES catalyst [1]	AALLME + GC-MS derivatization	Catalyzed derivatization enables ammonia quantification for air quality analysis
TPABr:isoamyl alcohol (1:6) [11]	DLLME + smartphone RGB colorimetry	Yellow DLL compound quantifies aqueous ammonium via digital colorimetry
DES-assisted Cr_2_AlC/Cr_2_CTx synthesis [13]	Green MAX-phase/MXene synthesis, gas-sensing test	Room-temperature NH_3_ gas detection demonstrated
Lactic acid:glucose:water NADES on laser-scribed carbon [35]	Direct laser scribing, chemometric optimization	Selective NH_3_ sensing (LOD 0.33%); applied to fish spoilage monitoring

## Data Availability

No new data were created or analyzed in this study. Data sharing is not applicable to this article.

## Abbreviations

The following abbreviations are used in this manuscript:

AALLME	Air-assisted liquid–liquid microextraction
AGREEprep	Analytical greenness metric for sample preparation
AIMD	Ab initio molecular dynamics
AMPH-Cl	Amino-2-propanol hydrochloride
ANN	Artificial neural network
[bmim][mesylate]	1-Butyl-3-methylimidazolium methanesulfonate
Cat	Catechol
ChCl	Choline chloride
CO_2_	Carbon dioxide
COSMO-RS	Conductor-like screening model for real solvents
COSMO-SAC	Conductor-like screening model segment activity coefficient
DES	Deep eutectic solvent
DLLME	Dispersive liquid–liquid microextraction
EACl	Ethanolamine hydrochloride
EG	Ethylene glycol
FTIR	Fourier-transform infrared spectroscopy
GA	Glycolic acid
GC-MS	Gas chromatography–mass spectrometry
GI	Guanidine isothiocyanate
Gly/GL	Glycerol
H_2_	Hydrogen
H_2_S	Hydrogen sulfide
HBA	Hydrogen-bond acceptor
HBD	Hydrogen-bond donor
HWA	Heterocyclic weak acid
IL	Ionic liquid
Im	Imidazole
[Im][NO_3_]	Imidazolium nitrate
[Im][SCN]	Imidazolium thiocyanate
LOQ	Limit of quantification
MAA	N-methylacetamide
MD	Molecular dynamics
MgCl_2_	Magnesium chloride
ML	Machine learning
MOF	Metal–organic framework
N_2_	Nitrogen
NADES	Natural deep eutectic solvent
NH_3_	Ammonia
NH_4_SCN	Ammonium thiocyanate
NMR	Nuclear magnetic resonance
NO	Nitric oxide
NO_2_	Nitrogen dioxide
NO_x_	Nitrogen oxides
OHP[5]	Per-hydroxylated pillar[5]arene
Phl	Phloroglucinol
PhOH	Phenol
PIL	Protic ionic liquid
PM2.5	Particulate matter (diameter ≤ 2.5 µm)
Res	Resorcinol
RGB	Red, green, blue
soft-SAFT	Soft statistical associating fluid theory
SO_x_	Sulfur oxides
TMPDA	Tetramethyl-propanediamine
TPABr	Tetrapropylammonium bromide
Tri/TrZ	Triazole
Tz	Tetrazole
UR	Urea
XYL	Xylitol
